# Associations between Statin/Omega3 Usage and MRI-Based Radiomics Signatures in Prostate Cancer

**DOI:** 10.3390/diagnostics11010085

**Published:** 2021-01-07

**Authors:** Yu Shi, Ethan Wahle, Qian Du, Luke Krajewski, Xiaoying Liang, Sumin Zhou, Chi Zhang, Michael Baine, Dandan Zheng

**Affiliations:** 1 Department of Biological Science, University of Nebraska Lincoln, Lincoln, NE 68588, USA; yu.shi@huskers.unl.edu (Y.S.); qian.du@huskers.unl.edu (Q.D.); 2 Department of Radiation Oncology, University of Nebraska Medical Center, Omaha, NE 68189, USA; EthanWahle@creighton.edu (E.W.); lkrajew1@alumni.nd.edu (L.K.); szhou@unmc.edu (S.Z.); 3 Department of Radiation Oncology, University of Florida Proton Institute, Jacksonville, FL 32206, USA; XLiang@floridaproton.org

**Keywords:** radiomics, prostate cancer, statin, omega-3, artificial intelligence, MRI

## Abstract

Prostate cancer is the most common noncutaneous cancer and the second leading cause of cancer deaths among American men. Statins and omega-3 are two medications recently found to correlate with prostate cancer risk and aggressiveness, but the observed associations are complex and controversial. We therefore explore the novel application of radiomics in studying statin and omega-3 usage in prostate cancer patients. On MRIs of 91 prostate cancer patients, two regions of interest (ROIs), the whole prostate and the peripheral region of the prostate, were manually segmented. From each ROI, 944 radiomic features were extracted after field bias correction and normalization. Heatmaps were generated to study the radiomic feature patterns against statin or omega-3 usage. Radiomics models were trained on selected features and evaluated with 500-round threefold cross-validation for each drug/ROI combination. On the 1500 validation datasets, the radiomics model achieved average AUCs of 0.70, 0.74, 0.78, and 0.72 for omega-3/prostate, omega-3/peripheral, statin/prostate, and statin/peripheral, respectively. As the first study to analyze radiomics in relation to statin and omega-3 uses in prostate cancer patients, our study preliminarily established the existence of imaging-identifiable tissue-level changes in the prostate and illustrated the potential usefulness of radiomics for further exploring these medications’ effects and mechanisms in prostate cancer.

## 1. Introduction

Approximately one in seven men in the US will be diagnosed with prostate cancer in their lives [1]. Prostate cancer is the most prevalent form of noncutaneous cancer and results in the second-highest number of cancer-related deaths behind only lung cancer. Since prostate cancer adversely affects so many people, it is essential to study and better understand the nature of its progression as well as patient prognosis after treatments, with the ultimate goal of improving treatment plans and their success.

Interesting correlations have been reported between prostate cancer and the use of statins and omega-3 fatty acids. Statins are a common heart medication, and omega-3 fatty acids are fish oil supplements. A study in 2018 found that there was a strong correlation between the use of statins and a reduced risk of prostate cancer metastasis, as well as a reduced risk of prostate cancer mortality [2]. Another study in 2018 focused on the effects of omega-3 fatty acids in the diet on prostate cancer tumor progression in mice and concluded that its use can inhibit the growth and development of tumor cells [3]. However, such associations have not been consistently observed among different studies, and a causative association was not fully established nor its mechanism uncovered [4,5,6,7,8,9,10,11,12,13,14]. These intriguing associations are complex and controversial, drawing active research for further elucidation. In this study, we explore the potential utility of radiomics along these efforts in analyzing the usage of these drugs among a prostate cancer patient population. 

Radiomics is a new field of medicine that involves extracting large amounts of quantitative data from medical images such as CT scans or MRIs [15,16]. The data extracted are termed radiomic features, and they can be anything from the intensity of pixels in an image to the shape and texture of regions of interest (ROIs). These features can also be extracted from derived images after applying image processing filters. Radiomics therefore provides additional data that are often not visible to the naked eye. Furthermore, using modern big data analytics, these large amounts of quantitative data can be studied across many patients to provide a new dimension of epidemiological data that relates to medical imaging phenotypes and the underlying biological characteristics. 

In prostate cancer, like in many other cancer sites, radiomics has found success in detecting and diagnosing tumors, characterizing index lesions, predicting tumor aggressiveness, evaluating treatment response and prognosis, and associating with tumor genomics [17,18,19,20,21,22,23,24,25,26,27]. However, to the best of our knowledge, radiomics has never been explored as a potential tool to investigate the relationship between medication exposure and prostate cancer. If radiomics signatures can be identified that associate with the use of these drugs, such radiomics tools can be utilized to further analyze the longitudinal trends between medication exposure and prostate cancer both during and after cancer development, based on available medical images. Using radiomics to study the correlation of statins and omega-3 in prostate cancer management adds a noninvasive, low-cost tool to assess any potential longitudinal tissue changes related to the drugs. Additionally, the presence of a defined radiomics signature that could differentiate patients that have been exposed to these medications and those who have not could help to validate the previous studies suggesting that these medications do indeed have tissue-level effects on the prostate gland as a whole as well as in prostate malignancy specifically [28,29]. Therefore, the aim of this study was to preliminarily explore the utility of radiomics on these novel investigations by discovering potential feature patterns that could offer further insight into the connections between prostate cancer and the use of these drugs through the lens of radiomics.

## 2. Materials and Methods 

### 2.1. Patient Selection and Clinical Data Collection

Male patients who developed prostate cancer and received radiation therapy treatment at our institution between 2007 and 2016 were retrospectively analyzed via electronic medical records for this study. To be included in the study, patients needed to have a definitive record of positive or negative use of statins and omega-3 supplements and a high-resolution T2-weighted MRI scan, at the time of diagnosis. A total of 91 patients met the criteria and were included in the study. In addition to the diagnostic high-resolution T2-weighted MRI scan, other information collected for the study included age, ethnicity, family history, pretreatment PSA, tumor stage, NCCN risk group, and tumor grade. All data collection was approved by the IRB of our institution (Protocol: 398-17-EP). The characteristics of the studied patient cohort are summarized in Table 1. The percentage of patients with certain clinical characteristics is also listed in Table 1 for each medication subgroup. Of the 91 patients, 42 used statins and 28 used omega-3. Among them, 13 patients used both medications. 

### 2.2. ROI Contouring and Image Processing

**Figure 1:**
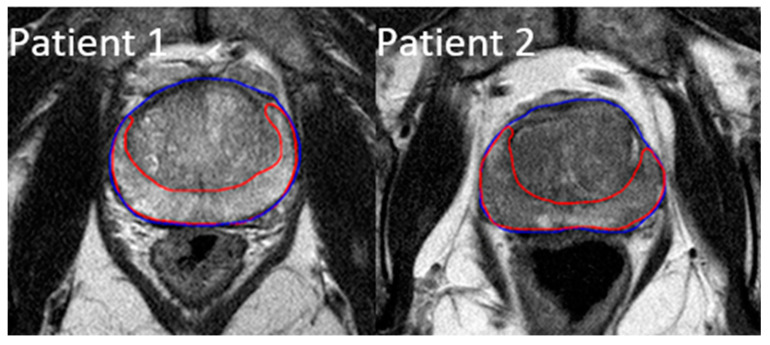
Example contours of the prostate region of interest (ROI) (blue) and the peripheral zone ROI (red).

All of the patients received a high-resolution T2-weighted MRI scan in the pelvic region at diagnosis. The images were taken using Philips Medical Systems Achieva 1.5 T MRI scanners with a 2 mm slice thickness. Using Velocity software (Varian Medical Systems, Palo Alto, CA, USA), each MRI scan was manually contoured on two separate ROIs: the prostate gland and the peripheral zone of the prostate. The whole prostate gland is the most common region reviewed by radiologists and also easy to contour for future studies, while the peripheral zone is where over 90% of prostate cancers develop [30]. Figure 1 shows the created contours on two example patients, with the prostate gland outlined in blue and its peripheral region outlined in red.

All of the images and segmented ROIs were then sent to 3D-Slicer v4.10 for image processing and radiomic feature extraction [31,32]. To avoid bias field distortions and data heterogeneity bias, a bias field correction using N4 and an image normalization using histogram matching were performed on all images [33].

### 2.3. Radiomic Feature Extraction

Radiomic feature extraction was performed using the radiomics module in 3D-Slicer v4.10 [32]. Feature extraction used a resampled voxel size of 2 × 2 × 2 mm^3^ and a fixed bin width of 25. A kernel size of 5 was used for Laplacian of Gaussian (LoG) feature extraction. For each ROI, a total of 944 radiomic features were extracted. All of the features are defined in compliance with the feature definitions according to the Imaging Biomarker Standardization Initiative (IBSI) [34]. They are divided into the original features (107 features), LoG features (93 features), and wavelet features (744 features). The original features contain 14 shape features, 18 first-order statistical features, 14 gray-level dependence matrix features, 24 gray-level co-occurrence matrix features, 16 gray-level run-length matrix features, 16 gray-level size zone matrix features, and 5 neighboring gray-tone difference matrix features. The LoG features are calculated on the derived images applying a LoG filter. The wavelet features are calculated on the derived images by applying either a high or low pass wavelet filter in each of the three cardinal dimensions, creating a total of 8 different filter combinations (HHH, HHL, HLL, HLH, LLL, LLH, LHL, and LHH). Because the shape features do not change on the derived images, the LoG and wavelet features do not include shape features.

### 2.4. Data Analysis

**Figure 2:**
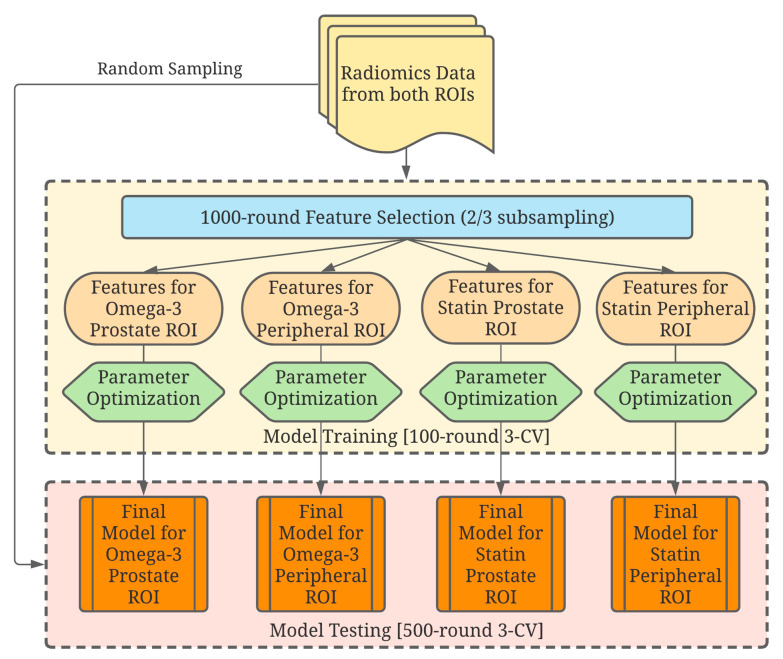
A schematic drawing of the data analysis workflow. Four independent combinations were analyzed, and their corresponding models were developed to study the effects of each drug on each region of interest (ROI).

Heatmaps were first generated based on each ROI to display the radiomic feature patterns for patients and their correlations with the use of statins or omega-3 supplements. Four combinations were made: Omega-3/Prostate, Omega-3/Peripheral, Statin/Prostate, and Statin/Peripheral. For each drug/ROI combination, a machine-learning model was created to select and analyze specific radiomic features that showed a correlation with drug usage. The model was trained and tested for its predictability with a workflow, depicted in Figure 2.

The 4 combinations of drug/ROI were studied independently in the data analysis workflow with the following steps. In the feature selection step, all patients were randomly sampled without replacement for 1000 rounds to use two-thirds of data for feature selection. Using the selected patients in each round, a univariate ANOVA analysis was first conducted on all radiomic features. Features with a false-discovery-rate-adjusted value of *p* < 0.5 were kept for the next step. The selected features with Pearson’s correlation coefficient > 0.8 were then grouped in clusters, and only the feature with the lowest *p*-value was kept in each cluster to further reduce the dimension [35]. A sequential floating-forward method was then utilized to select features, which gave the highest area-under-the-curve (AUC) value with a gradient boost machine-learning model [36]. Only the features that could improve the AUC by more than 0.01 in each round were kept. All features were ranked by the frequency of those features selected in all 1000 rounds of resampling and the top 10 features were selected.

Model fitting and validation applied a gradient boost machine-learning model [36] using the default gradient-boosting machine classifier integrated in mlr (v2.18.0) (R Foundation for Statistical Computing, Vienna, Austria) [37]. The best number of features in a model was balanced between high bias (an underfitted model) and high variance (an overfitted model). For each round of model fitting, the total search space is 8 × 4 × 3 × 3 × 3 × 3 = 2596, including the number of top features from 3 to 10 features (8), the number of trees (4), shrinkage (3), interaction depth (3), the minimum number of training set samples in a node to commence splitting (3), and bag fraction (3). A 500-round randomly sampled 3-fold cross-validation was applied, yielding 1500 validation tests for each drug/ROI combination. For each test, a receiver operating characteristic (ROC) analysis was conducted with the AUC and precision-recall AUC (PRAUC) values calculated. All data analysis was performed using R (version 4.0.2).

## 3. Results

**Figure 3:**
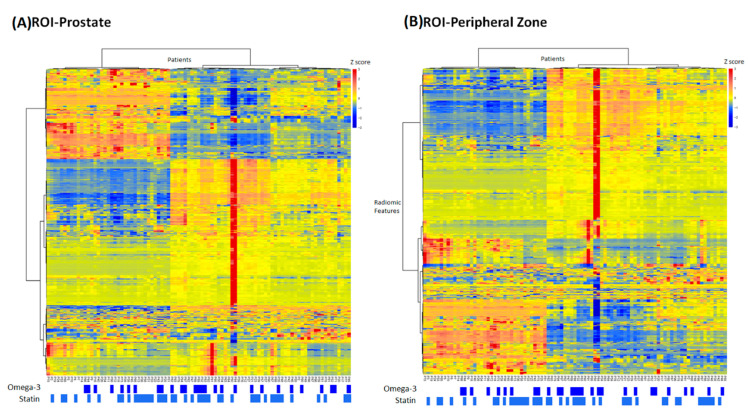
Heatmaps showing the radiomics feature pattern across the patient population with the usage of omega-3 and statin for (**A**) the prostate ROI and (**B**) the peripheral ROI.

In the heatmaps shown in Figure 3, the studied patients were clustered into similar radiomic expression patterns of the prostate gland and the peripheral region of the prostate. The information regarding statin use and omega-3 use is also included. Two outlier patients were observed with extreme feature z-scores, but excluding them did not affect feature selection or model performance (data not shown), likely due to the robust resampling procedures performed in data analysis. 

**Figure 4:**
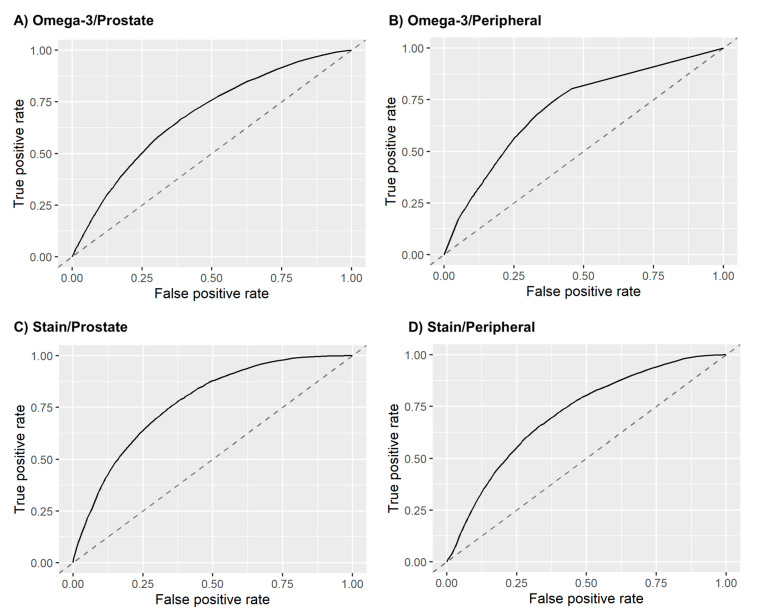
Average ROC curves of the radiomics model for (**A**) omega-3 usage prediction based on prostate ROI, (**B**) omega-3 usage prediction based on peripheral ROI, (**C**) statin usage prediction based on prostate ROI, and (**D**) statin usage prediction based on peripheral ROI. The ROC curves are averaged from the 1500 validation test sets.

**Figure 5:**
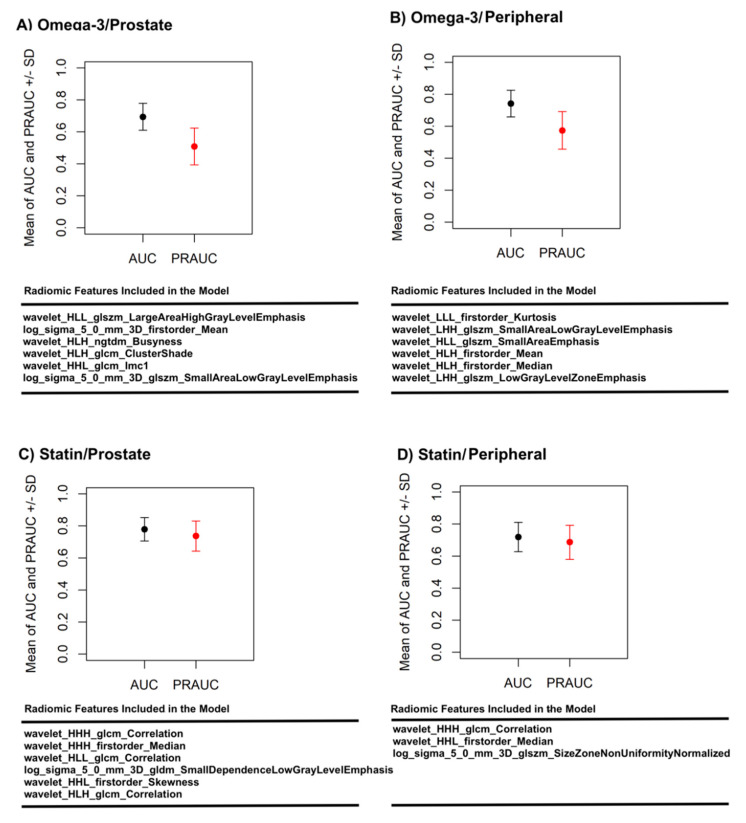
Area under the curve (AUC) and precision AUC (PRAUC) achieved on the 1500 validation sets by the radiomics model for (**A**) omega-3 usage prediction based on prostate ROI, (**B**) omega-3 usage prediction based on peripheral ROI, (**C**) statin usage prediction based on prostate ROI, and (**D**) statin usage prediction based on peripheral ROI. The radiomic features used in each model are also listed.

Following the data analysis pipeline described in the Methods section (Section 2), a model was then constructed and tested for each of the four drug/ROI combinations: omega-3/prostate ROI (six features), omega-3/peripheral ROI (six features), statin/prostate ROI (six features), and statin/peripheral ROI (six features). On the 1500 validation datasets from the 500-round resampled threefold cross-validation, the models achieved an average (standard deviation) AUCs of 0.70 (± 0.08) for omega-3/prostate, 0.74 (± 0.08) for omega-3/peripheral, 0.78 (± 0.07) for statin/prostate, and 0.72 (± 0.09) for statin/peripheral. Figure 4 plots the average ROC curves from the validation analyses for these models, and Figure 5 shows the validation AUC and PRAUC achieved by each model. The selected radiomic features used in each model are also shown in Figure 5.

## 4. Discussion

Radiomics is a novel quantitative imaging approach that involves extracting a large number of features from medical images and developing predictive models based on high-dimensional data analysis across large groups of patients. The underlying assumption is that the modern high-resolution cross-sectional medical images contain information on biology and physiology that is not fully extracted or utilized by the conventional qualitative, single-patient approach. Combined with machine learning on populations of patients to uncover the otherwise hidden information from medical images, radiomics has been shown to facilitate the detection, diagnosis, and prognosis of many diseases, especially cancer [15,16,38,39].

Prostate cancer is the most prevalent noncutaneous male cancer and is the second leading cause of cancer death for men in the United States [1]. Intragland and intratumor heterogeneity have also been well observed for prostate cancer. Based on MRI used in the routine management of prostate cancer, radiomics is well posited to study these heterogeneities as well as to assess the heterogeneity among different patients. While still in an early stage of development as a discipline, radiomics has found success in prostate cancer diagnosis, risk characterization, genomic association, and prognosis prediction, offering a noninvasive and repeatable approach in these applications [18,19,20,21,22,24,25,27,40]. With recent research, epidemiological, and clinical development in prostate cancer, risk stratification has become an increasingly central theme in prostate cancer management. Risk identification and prediction play a critical role in personalized clinician–patient decision making. Radiomics therefore provides a potential tool for exploring the intricate interplays between medications and health supplements such as statin and omega-3 and prostate cancer, which are currently active areas of risk investigations.

In this study, we explored the relationship between radiomics and the use of statins and omega-3 supplements for a cohort of 91 prostate cancer patients. This study used radiomic data based on high-resolution T2-weighted MRI, the most used type of medical imaging technique for prostate cancer diagnosis. On this topic, radiomics is a novel approach and can be utilized as an effective method to determine possible prostate tissue effects related to medications with imaging rather than with more invasive/expensive methods such as biopsies. Two different ROIs were investigated, including the whole prostate gland and the peripheral region of the prostate. The whole prostate gland is the most common region reviewed by radiologists, while the peripheral zone is where over 90% of prostate cancers develop [30], so the two ROIs were separately analyzed to see if there was a performance difference. Through this study, we identified specific radiomics signatures that correlated with the use of these drugs and presented machine-learning models based on these signatures. These signatures were ultimately able to successfully predict the use of both of these drugs for a given patient with prostate cancer. Between the two segmented regions, the prostate gland showed a slightly lower AUC than the peripheral zone (0.70 vs. 0.74) for the omega-3 models but a slightly higher AUC (0.78 vs. 0.72) for the statin models. On the other hand, the statin/peripheral model used only three radiomic features, while the other three models used six features. The current study preliminarily establishes radiomics signatures that help to validate the previously found tissue-level effect of the medications on the prostate and could allow us to further investigate the longitudinal tissue effects of statins and omega-3 over the course of prostate cancer development and growth using this new dimension of data. 

There are some limitations to this work. The study was a single-institution, retrospective study, with a relatively small number of patients (91 patients) meeting the selection criteria. To maximize the available data and avoid overfitting, we utilized cross-validation of a large number of random repetitions, and the number of radiomic features in the final models was also limited to 3–6 features. For omega-3, the data were more unbalanced, which could also lead to overfitting. To study its effect, we calculated both the AUC and PRAUC for each model. As expected, bigger drops were observed in the PRAUCs for the omega-3 models. To further assess the chance of overfitting despite the above mitigating measures, we also performed a control experiment with a third medication, angiotensin-converting enzyme inhibitors (ACEI). ACEI is a type of renin–angiotensin system (RAS) inhibitor and is commonly used to treat hypertension. A few studies have analyzed the association between ACEI and prostate cancer [41,42]. These studies tend to suggest that ACEI has no effect on prostate cancer development or progression. In our cohort, 35 patients (38.5%) reported using ACEIs. Applying our radiomics data analysis workflow through the ACEI usage data, we achieved an average AUC of 0.58 and an average PRAUC of 0.49 with the ACEI/prostate model (four features) and 0.54 and 0.52 with the ACEI/peripheral model (six features). The superior model performances by the statin and omega-3 models compared with the models of the control medication, ACEI, further confirmed the validity of our radiomic findings on the two investigated medications.

Additionally, most of the patients in the dataset had their primary care physicians outside of our institution, so we were unable to have access to the initial lipid profiles to assess the reasoning behind their use of these medications or any related medical care differences that these patients may have. In our study, we individually studied the four drug/ROI combinations. The medication overlap on the 13 patients that used both medications was not considered, assuming that the possible mechanisms of prostate cancer for the two medications are independent of each other. We also did not match the other patient characteristics for the positive and negative subgroups of each medication. These choices were made so that the sample size could be maximized for the study. While biases could possibly be introduced, they are unlikely because there is currently no literature suggesting a correlation between the mechanisms of the two medications and prostate cancer, and the patient subgroups are largely balanced on other characteristics, as shown in Table 1. Furthermore, in this study, we analyzed radiomics based on imaging data only at a single time point; the time of diagnosis and the effect of varying lengths of medication usage before this time point were not investigated. While this could have acted as a confounding factor in this study, the promising findings of this preliminary study could guide future endeavors to use the radiomics tool for exploring the longitudinal tissue effects related to these medication uses in the course of prostate cancer development and progression.

## 5. Conclusions

For the first time, the potential of radiomics in investigating drug/cancer interactions was investigated in this study of statin and omega-3 in prostate cancer patients. While needing further large-scale validations, encouraging findings from this study indicate that radiomics supports tissue-level effects of these medications and that radiomics can be a useful tool for future studies on the longitudinal tissue effects related to these medications in the course of prostate cancer development and progression. Either the whole prostate gland or the peripheral region could be used as an appropriate volume for future radiomics studies on this topic.

## Figures and Tables

**Table 1:** Patient Demographics.

Parameters	Medication Usage				All	
	Statin		Omega-3			
	Yes (%)	No (%)	Yes (%)	No (%)	N	%
Total Number of Patients	42 (46.2%)	49 (53.8%)	28 (30.8%)	63 (69.2%)	91	100%
Age at Diagnosis (median(range))	66 (54–83)y	67 (54–84)y	65 (57–82)y	68 (54–84)y	67 (54–84)y	
Race						
White	85.7%	85.7%	82.1%	87.3%	78	85.7%
Non-White	14.3%	14.3%	17.9%	12.7%	13	14.3%
Family History of Prostate Cancer						
No	78.6%	75.5%	75.0%	76.2%	70	76.9%
Yes	21.4%	24.5%	25.0%	23.8%	21	23.1%
NCCN Risk Group						
Low/Favorable Intermediate	14.3%	14.3%	17.9%	12.7%	13	14.3%
Unfavorable Intermediate	21.4%	18.4%	17.9%	20.6%	18	19.8%
High	21.4%	24.5%	21.5%	23.8%	21	23.1%
Very High	42.9%	42.8%	42.9%	42.9%	39	42.9%
Gleason Grade, Primary						
3 (4+3)	40.5%	38.8%	42.9%	38.1%	36	39.6%
4 (8)	54.8%	53.1%	50.0%	55.6%	49	53.8%
5 (9, 10)	4.8%	8.2%	7.1%	6.3%	6	6.6%
Gleason Grade, Secondary						
3 (4+3)	23.8%	22.4%	17.9%	25.4%	21	23.1%
4 (8)	23.8%	24.5%	28.6%	22.2%	22	24.2%
5 (9, 10)	52.4%	53.1%	53.6%	52.4%	48	52.8%
cT						
cT1c	26.2%	26.5%	21.4%	28.6%	24	26.4%
cT2						
cT2a	16.7%	18.4%	21.5%	16.0%	16	17.6%
cT2b	14.3%	8.2%	14.3%	9.5%	10	11.0%
cT2c	7.1%	10.2%	10.7%	7.9%	8	8.8%
cT3	21.4%	22.4%	17.9%	23.8%	20	22.0%
cT3a	14.3%	14.3%	14.3%	14.3%	13	14.3%
cT3b						
cN						
0	88.1%	85.7%	82.1%	88.9%	79	86.8%
1	11.9%	14.3%	17.9%	11.1%	12	13.2%
PSA						
Pretreatment PSA (median(range))	9.69 (1.31–52.19)	10.00 (1.39–59.99)	10.73 (2.00–52.19)	9.59(1.31–59.99)	9.69 (1.31–59.99)	

## Data Availability

The data presented in this study are available on request from the corresponding author.
